# A GPU-Oriented Onboard Imaging Framework for High-Resolution Sliding Spotlight SAR on an Embedded GPU Platform

**DOI:** 10.3390/s26144592

**Published:** 2026-07-20

**Authors:** Ziyang Dai, Jian Liu, Zhanyang Ai, Yang Liu, Ziyan Wang, Zongwei Zhu

**Affiliations:** 1 School of Software Engineering, University of Science and Technology of China, Hefei 230026, China; daiziyangcn@mail.ustc.edu.cn; 2 Aerospace Information Research Institute, Chinese Academy of Sciences, Beijing 100094, China; 3 Anhui Zhongke Miwei Technology Co., Ltd., Hefei 230031, China; 4 Suzhou Institute for Advanced Research, University of Science and Technology of China, Suzhou 215123, China

**Keywords:** Synthetic Aperture Radar, sliding spotlight SAR, onboard SAR processing, embedded GPU, Jetson AGX Orin, GPU acceleration

## Abstract

In conventional spaceborne Synthetic Aperture Radar (SAR) systems, raw echo data are usually downlinked to ground stations for image formation, resulting in substantial communication burden and processing delay. Although onboard SAR imaging can alleviate this problem, onboard processors are constrained by power consumption, memory capacity, thermal dissipation, and physical size. Therefore, low-power embedded GPU platforms have become a practical choice for onboard SAR processing. Existing onboard processing is mainly suitable for relatively low-complexity imaging modes and algorithms, while high-resolution sliding spotlight SAR requires more accurate frequency-domain processing due to its extended azimuth bandwidth, strong range–azimuth coupling, and nonlinear range cell migration. The ω-k algorithm is well-suited for such high-resolution imaging scenarios, but its large-scale FFTs, phase compensation, and Stolt interpolation impose significant pressure on the memory capacity, memory bandwidth, and computational resources of embedded GPU platforms. To address these challenges, this paper presents a GPU-oriented ω-k imaging framework for high-resolution sliding spotlight SAR on the Jetson AGX Orin embedded platform. The proposed framework formulates a complete sliding spotlight ω-k processing flow and develops GPU-oriented optimization strategies for efficient execution on the embedded GPU platform. Specifically, hybrid data partitioning is designed to adapt memory access patterns to range- and azimuth-dominant stages, an asynchronous multi-stream pipeline with reusable GPU buffers is introduced to overlap data movement and computation, and customized kernels are developed for Stolt interpolation and FFT-related spectral centering. Experiments on simulated sliding spotlight SAR data demonstrate that the proposed method achieves well-focused imaging results with consistent impulse response characteristics. For a 32,768× 32,768 simulated SAR dataset, the proposed implementation achieves an end-to-end imaging time of 32.17 s on Jetson AGX Orin. These results demonstrate the feasibility of the proposed framework for onboard high-resolution SAR imaging on an embedded GPU platform.

## 1. Introduction

Synthetic Aperture Radar (SAR) is a mature microwave remote sensing imaging technology that offers all-weather and all-time operational capability [1,2]. Owing to its strong penetration ability and high resolution imaging performance, SAR has been widely applied in Earth observation, resource exploration, disaster monitoring, and military reconnaissance [1,3]. In conventional spaceborne SAR systems, satellites are typically responsible only for raw echo data acquisition and basic preprocessing, after which the unfocused data are downlinked to ground stations for imaging processing. However, this traditional processing paradigm is increasingly challenged in modern and future SAR applications.

On the one hand, continuous improvements in SAR system resolution have led to a rapid growth in data volume. Limited by the bandwidth of satellite-to-ground communication links and the availability of downlink time windows, large amounts of raw echo data cannot be transmitted to the ground in a timely and complete manner. On the other hand, the requirement to perform imaging processing on the ground inevitably introduces significant latency, making this paradigm unsuitable for time-sensitive applications such as disaster emergency response, dynamic target monitoring, and rapid reconnaissance missions [4].

In recent years, the rapid advancement of embedded computing platforms has enabled low-power, high-performance devices to execute complex algorithms under strict energy constraints [5]. As a result, computationally intensive tasks that were traditionally confined to ground processing are increasingly becoming feasible on board [6,7]. Against this backdrop, satellite systems are evolving toward constellation-based, intelligent, and rapid response architectures [8]. Migrating SAR imaging from ground systems to onboard platforms has therefore emerged as a key technological approach to alleviate satellite-to-ground data transmission pressure and reduce imaging latency [7]. Onboard SAR processing can significantly reduce the volume of downlinked data and enable real-time or near real-time imaging, which is of great importance for disaster monitoring, military reconnaissance, and dynamic target surveillance [9].

Despite these advantages, onboard embedded GPUs face stringent resource constraints compared with data center–class GPUs, including limited memory capacity, reduced memory bandwidth, and constrained computational resources [10]. Under these limitations, current onboard SAR processing capabilities are primarily focused on relatively low-complexity imaging modes such as stripmap, where the Doppler bandwidth is moderate and range cell migration (RCM) can be approximated using simple models [2]. In contrast, high-resolution imaging modes, including sliding spotlight and spotlight SAR, involve significantly expanded azimuth bandwidth, strong range–azimuth coupling, and highly nonlinear RCM behavior, which substantially increase both computational and memory demands [11,12,13,14,15,16]. As a result, these advanced imaging modes are still predominantly processed on the ground in most existing satellite systems. Consequently, enabling efficient onboard processing for high-resolution SAR imaging remains a critical challenge. Addressing this issue requires not only algorithmic optimization to reduce computational complexity, but also careful parallel implementation tailored to embedded GPU architectures.

The ω-K Algorithm (ωKA) is one of the most accurate frequency-domain SAR imaging algorithms for high resolution applications [2,17,18]. By transforming the received echo data into the two-dimensional wavenumber domain, ωKA directly models the wave propagation geometry and avoids some of the low-order approximations commonly adopted in conventional stripmap-oriented methods. Through a sequence of large-scale Fast Fourier Transforms (FFTs), reference function phase compensation, and Stolt interpolation, the algorithm can accurately correct range migration and achieve high-quality focusing. Owing to its rigorous physical foundation and superior imaging accuracy, ωKA has become an important solution for high-resolution SAR imaging, especially in scenarios involving large bandwidth, long synthetic aperture, or strong range–azimuth coupling.

To enable efficient onboard execution under constrained resources, the sliding spotlight ω-k algorithm is first organized into a complete and structured processing flow, including range frequency preprocessing, azimuth spectral reshaping, wavenumber domain phase compensation, Stolt interpolation, and azimuth postprocessing. Although these stages have different signal-processing purposes, their underlying computations can be summarized into three main operation types: repeated large-scale FFT and IFFT operations, pointwise complex multiplications for phase compensation, and a nonuniform Stolt interpolation step. These operations are highly structured and largely data-parallel, providing the algorithmic basis for mapping the ω-k imaging procedure onto an embedded GPU platform.

Based on the computational characteristics of the above stages, GPU-oriented implementation strategies are then developed. For range- and azimuth-dominant FFT processing stages, a hybrid data partitioning strategy is adopted to improve memory access locality and reduce unnecessary full-matrix data reorganization. To further improve sustained throughput during block-wise execution, an asynchronous multi-stream pipeline with reusable GPU buffers is introduced to overlap data movement and GPU computation. In addition, the Stolt interpolation stage is implemented using a customized CUDA kernel with fixed-length sinc interpolation, while a phase-modulation-based spectral centering strategy is used to avoid explicit data reordering in FFT and IFFT operations. By matching the implementation strategy to the computational characteristics of each processing stage, the proposed framework improves memory efficiency, parallel execution efficiency, and overall throughput on the Jetson AGX Orin embedded platform.

The main contributions of this paper can be summarized as follows:

(1) An efficient ω-k imaging framework for sliding spotlight SAR is developed. Starting from the signal model, the complete imaging procedure is systematically formulated, including range frequency preprocessing, azimuth domain spectral reshaping, two-dimensional wavenumber domain focusing, and azimuth postprocessing. The proposed formulation enables accurate phase compensation and full-aperture focusing under sliding spotlight acquisition, effectively handling the extended aperture and nonlinear range cell migration effects.

(2) A GPU-oriented algorithm restructuring strategy is proposed to enable efficient execution under memory bandwidth constraints. By analyzing the alternating range–azimuth computation pattern in the ω-k pipeline, a hybrid data partitioning scheme is designed to dynamically match the dominant computation direction. Combined with asynchronous multi-stream pipeline parallelism and buffer reuse mechanisms, the proposed approach effectively overlaps data transfer and computation, significantly improving memory access efficiency and overall throughput on embedded GPU platforms.

(3) A set of computation-level optimizations is developed for key processing stages. These include a customized GPU implementation of Stolt interpolation with two-dimensional thread mapping, constant precomputation, and loop unrolling, as well as a frequency-domain centering strategy based on phase modulation that avoids explicit data reordering. These optimizations transform memory-bound operations into compute-efficient kernels, leading to improved parallel efficiency and reduced execution overhead.

(4) The proposed method is implemented and evaluated on a Jetson AGX Orin embedded GPU platform. Experimental results demonstrate that the proposed approach achieves high-quality SAR imaging while significantly improving computational efficiency under strict power, memory, and bandwidth constraints, validating its feasibility for onboard high-resolution SAR processing on an embedded GPU platform.

## 2. Sliding Spotlight ω-k Imaging Algorithm

### 2.1. Sliding Spotlight SAR Geometry and Signal Model

The synthetic aperture length in sliding spotlight mode is significantly larger than that in stripmap mode, resulting in an extended target dwell time and improved azimuth resolution [11,12,13,15,19]. However, unlike conventional spotlight mode, in which the antenna beam continuously stares at the scene center during the aperture time, sliding spotlight mode does not maintain full-time beam pointing toward the scene center. In stripmap mode, the antenna boresight remains fixed with respect to the platform, whereas in spotlight mode, it is continuously steered toward the scene center. Sliding spotlight mode can be regarded as an intermediate case between these two modes, where the antenna beam is steered slowly in the azimuth direction. In particular, the antenna beam center is continuously directed toward a virtual reference point located outside the imaged scene.

**Figure 1:**
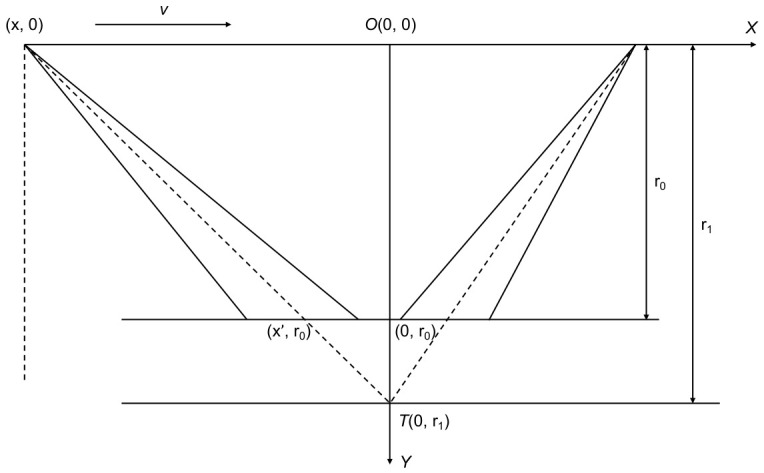
Imaging geometry of sliding spotlight SAR.

The imaging geometry of sliding spotlight SAR is illustrated in Figure 1. The SAR platform moves along the azimuth direction, defined as the *X*-axis, with a constant velocity *v*. In the corresponding slant plane, the cross-track direction is defined as the *Y*-axis. During data acquisition, the antenna beam center is continuously steered toward the virtual point *T*. Let $r_{0}$ denote the minimum slant range from the platform to the scene reference point, and let $r_{1}$ denote the minimum slant range from the platform to the virtual point *T*. Then, the instantaneous slant range from the platform to the scene reference point is given by

(1) $$R\left(\eta \right)=\sqrt{v^{2}\eta^{2}+r_{0}^{2}}$$

where η denotes the slow time, and η=0 corresponds to the instant of closest approach to the scene reference point.

For a point target, the received baseband echo in sliding spotlight mode can be expressed as

(2) $$s\left(\tau ,\eta \right)=\sigma \;w_{r}\;\left(\tau -\frac{2R(\eta )}{c}\right)w_{a}\left(\eta -\eta_{c}\right)\exp\;\left[j\pi K_{\tau }{\left(\tau -\frac{2R(\eta )}{c}\right)}^{2}\right]\exp\;\left[-j\frac{4\pi }{\lambda }R\left(\eta \right)\right]$$

where τ denotes the fast time, σ denotes the complex reflectivity of the point target, $w_{r}\left(\cdot \right)$ and $w_{a}\left(\cdot \right)$ denote the range and azimuth window functions, respectively, $\eta_{c}$ is the azimuth time at which the target is illuminated by the beam center, $K_{\tau }$ denotes the range chirp rate of the transmitted linear frequency modulation (LFM) pulse, λ is the radar wavelength, and *c* is the speed of light.

**Figure 2:**
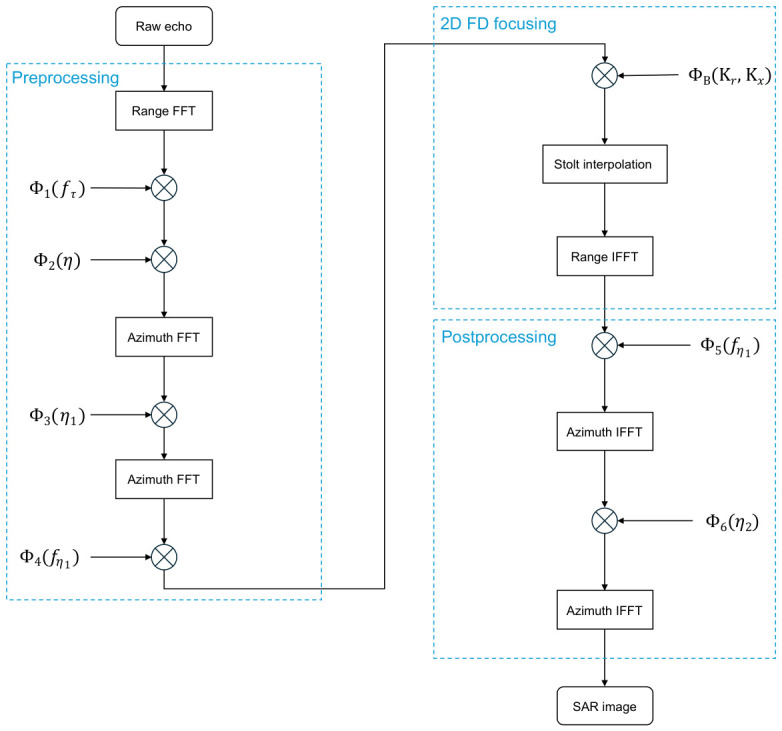
Flowchart of sliding spotlight SAR imaging processing.

Based on the signal model in (2), the complete imaging procedure for sliding spotlight SAR can be derived in a structured manner. The corresponding processing flowchart is shown in Figure 2. Starting from the raw echo data, the algorithm first performs range frequency preprocessing and azimuth domain preprocessing to prepare the signal for full-aperture processing. It then proceeds to two-dimensional frequency-domain focusing in the wavenumber domain, where bulk compression and Stolt interpolation are applied to achieve accurate phase compensation. Finally, azimuth domain postprocessing is conducted to restore the azimuth support and obtain the focused image. The flowchart summarizes the transformations of the signal across different domains and provides a clear representation of the overall imaging pipeline.

### 2.2. Range Frequency Preprocessing and Azimuth Domain Preprocessing

Starting from the raw echo s(τ,η) in (2), a Fourier transform is first performed along the range direction:

(3) $$S_{0}\left(f_{\tau },\eta \right)=F_{\tau }\left\{s(\tau ,\eta )\right\}$$

where $F_{\tau }\left\{\cdot \right\}$ denotes the Fourier transform with respect to the fast time variable τ, and $f_{\tau }$ denotes the corresponding range frequency variable.

According to the raw echo model in (2), the range LFM phase term can be written as

(4) $$\exp[j\pi K_{\tau }{\left(\tau -\frac{2R(\eta )}{c}\right)}^{2}]$$

Under the stationary phase approximation, its Fourier transform with respect to τ contains a range frequency quadratic phase term:

(5) $$F_{\tau }\{\exp[j\pi K_{\tau }{\left(\tau -\frac{2R(\eta )}{c}\right)}^{2}]\}\propto \exp\left(-j\frac{\pi }{K_{\tau }}f_{\tau }^{2}\right)\exp\left(-j\frac{4\pi }{c}f_{\tau }R\left(\eta \right)\right)$$

Here, ∝ indicates equality up to a constant amplitude and phase factor that does not affect the subsequent phase compensation. Therefore, a range frequency reference function is introduced to compensate the quadratic phase term:

(6) $$\Phi_{1}\left(f_{\tau }\right)=\exp\left(j\frac{\pi }{K_{\tau }}f_{\tau }^{2}\right)$$

The signal after range frequency preprocessing is then written as

(7) $$S_{1}\left(f_{\tau },\eta \right)=S_{0}\left(f_{\tau },\eta \right)\;\Phi_{1}\left(f_{\tau }\right)$$

After range frequency preprocessing, azimuth domain compensation is applied to reshape the azimuth spectrum under sliding spotlight acquisition. In sliding spotlight SAR, the antenna beam is steered along the azimuth direction, which introduces an approximately linear variation of the Doppler centroid with respect to azimuth slow time. This relationship can be expressed as

(8) $$f_{\mathrm{dc}}\left(\eta \right)\approx K_{\mathrm{dc}}\eta$$

where $K_{\mathrm{dc}}$ is the Doppler centroid change rate induced by beam steering. This Doppler centroid variation enlarges the total azimuth Doppler bandwidth and must be compensated before full-aperture wavenumber domain focusing.

Since a quadratic phase signal has a linearly varying instantaneous frequency, the spectral analysis (SPECAN)-based two-step processing (TSP) principle can be used for azimuth spectral reshaping. Specifically, the instantaneous frequency of

(9) $$\exp(j\pi K_{\mathrm{dc}}\eta^{2})$$

is

(10) $$\frac{1}{2\pi }\frac{d}{d\eta }\left(\pi K_{\mathrm{dc}}\eta^{2}\right)=K_{\mathrm{dc}}\eta$$

Therefore, a quadratic phase factor is introduced in the original azimuth slow time domain:

(11) $$\Phi_{2}\left(\eta \right)=\exp\;\left(j\pi K_{\mathrm{dc}}\eta^{2}\right)$$

The signal after the first azimuth quadratic phase compensation is

(12) $$S_{2}\left(f_{\tau },\eta \right)=S_{1}\left(f_{\tau },\eta \right)\;\Phi_{2}\left(\eta \right)$$

To enlarge the azimuth spectral support required by sliding spotlight processing, the azimuth dimension is zero-padded from $N_{a0}$ to $N_{a}$, where $N_{a0}$ and $N_{a}$ denote the numbers of azimuth samples before and after zero-padding, respectively. A new azimuth time variable $\eta_{1}$ is then introduced for the padded signal, denoted by

(13) $${\tilde{S}}_{2}\left(f_{\tau },\eta_{1}\right)$$

The zero-padding operation enlarges the discrete azimuth support and prepares the signal for the subsequent SPECAN-based spectral mapping.

Following the SPECAN principle, the azimuth Doppler frequency and the new azimuth time variable satisfy the approximate mapping relation

(14) $$f_{\eta }=K_{\mathrm{dc}}\eta_{1}$$

This relation indicates that the Doppler frequency variable can be mapped to a scaled azimuth time variable after quadratic phase modulation and Fourier transformation. Accordingly, the equivalent azimuth sampling frequency after the SPECAN/TSP mapping is given by

(15) $${\mathrm{PRF}}_{new}=K_{\mathrm{dc}}T_{a}=K_{\mathrm{dc}}\frac{N_{a}}{\mathrm{PRF}}$$

where $T_{a}=N_{a}/\mathrm{PRF}$ denotes the azimuth time span after zero-padding, and PRF is the original pulse repetition frequency.

After zero-padding, another quadratic phase factor is introduced in the new azimuth time domain:

(16) $$\Phi_{3}\left(\eta_{1}\right)=\exp\;\left(j\pi K_{\mathrm{dc}}\eta_{1}^{2}\right)$$

The Fourier transform of a quadratic phase function produces another quadratic phase term in the frequency domain. For the SPECAN operation, the corresponding frequency-domain quadratic phase compensation can be written as

(17) $$\Phi_{4}\left(f_{\eta_{1}}\right)=\exp\;\left(j\frac{\pi }{K_{\mathrm{dc}}}f_{\eta_{1}}^{2}\right)$$

Here, $f_{\eta_{1}}$ denotes the azimuth frequency variable corresponding to $\eta_{1}$.

Combining the above operations, the output of the preprocessing stage can be written in compact form as

(18) $$S_{A}\left(f_{\tau },f_{\eta_{1}}\right)=F_{\eta_{1}}\left\{F_{\eta_{1}}\left[{\tilde{S}}_{2}\left(f_{\tau },\eta_{1}\right)\;\right]\Phi_{3}\left(\eta_{1}\right)\right\}\Phi_{4}\left(f_{\eta_{1}}\right)$$

where $F_{\eta_{1}}\left\{\cdot \right\}$ denotes the Fourier transform with respect to $\eta_{1}$.

The above operations jointly accomplish two tasks. First, they reshape the azimuth signal into a form suitable for subsequent full-aperture frequency domain focusing. Second, they expand the equivalent azimuth spectral support so that the following wavenumber domain processing can be performed without loss of useful spectral information.

### 2.3. 2-D Frequency Domain Focusing

After the preprocessing stage, the signal is focused in the 2-D frequency domain. The processing is described in the wavenumber domain. Let $K_{r}$ denote the range wavenumber, $K_{x}$ denote the azimuth wavenumber, $K_{rc}$ denote the reference range wavenumber, and $r_{c}$ denote the reference slant range. The range wavenumber and the azimuth wavenumber are defined as

(19) $$K_{r}=\frac{4\pi (f_{0}+f_{\tau })}{c}=K_{rc}+\frac{4\pi f_{\tau }}{c}$$

and

(20) $$K_{x}=\frac{2\pi f_{\eta_{1}}}{v}$$

where $f_{0}$ is the carrier frequency, $f_{\tau }$ is the range frequency, *v* is the platform velocity, and *c* is the speed of light. The reference range wavenumber is given by

(21) $$K_{rc}=\frac{4\pi f_{0}}{c}$$

The bulk compression phase function is defined as

(22) $$\Phi_{B}\left(K_{r},K_{x}\right)=\exp\;\left(jr_{c}\sqrt{K_{r}^{2}-K_{x}^{2}}\right)\exp\;\left[-j\left(K_{r}-K_{rc}\right)r_{c}\right]$$

Accordingly, the bulk compressed signal is

(23) $$S_{B}\left(K_{r},K_{x}\right)=S_{A}\left(K_{r},K_{x}\right)\;\Phi_{B}\left(K_{r},K_{x}\right)$$

In (22), the first exponential term performs bulk phase compensation in the 2-D wavenumber domain, whereas the second exponential term removes the dominant reference phase associated with the reference slant range $r_{c}$. As a result, the target located at the reference position is well-focused. However, targets deviating from the reference position still contain residual space-variant phase modulation. Therefore, Stolt interpolation is further applied to remove the residual phase error and complete the range migration correction.

Following the standard ω-k formulation, the Stolt mapping is expressed in the frequency domain as

(24) $$f_{\tau 1}=\sqrt{{\left(f_{\tau }+f_{0}\right)}^{2}-\frac{c^{2}f_{\eta_{1}}^{2}}{4v^{2}}}$$

where $f_{\tau 1}$ denotes the new range frequency after Stolt interpolation. Using the definitions of $K_{r}$ and $K_{x}$ in (19) and (20), (24) can be equivalently expressed in the wavenumber domain as

(25) $$K_{y}=\sqrt{K_{r}^{2}-K_{x}^{2}}$$

where $K_{y}$ denotes the remapped range wavenumber after Stolt interpolation.

Since the inverse Fourier transform in the range direction requires samples on a uniformly spaced $K_{y}$ grid, the signal originally sampled on the $K_{r}$ grid must be resampled onto the $K_{y}$ grid. In the discrete implementation, the output samples are placed on a uniform $K_{y}$ grid. Therefore, for each desired output sample $K_{y}$ and azimuth wavenumber $K_{x}$, the corresponding input range wavenumber is obtained by the inverse Stolt mapping:

(26) $$K_{r}^{\mathrm{in}}\left(K_{y},K_{x}\right)=\sqrt{K_{y}^{2}+K_{x}^{2}}$$

The interpolation relation used in the implementation is then written as

(27) $$S_{C}\left(K_{y},K_{x}\right)=S_{B}\;\left(K_{r}^{\mathrm{in}}\left(K_{y},K_{x}\right),K_{x}\right)$$

This equation indicates that, for each azimuth wavenumber $K_{x}$, the signal is interpolated along the range wavenumber direction from the nonuniform input location $K_{r}^{\mathrm{in}}\left(K_{y},K_{x}\right)$ to the uniform output grid $K_{y}$.

The original range wavenumber grid is uniformly sampled according to (19). Therefore, the fractional interpolation index corresponding to $K_{r}^{\mathrm{in}}$ is calculated as

(28) $$n_{y}=\frac{K_{r}^{\mathrm{in}}\left(K_{y},K_{x}\right)-K_{rc}}{4\pi }\frac{c}{f_{s}}N_{r}+\frac{N_{r}}{2}$$

where $f_{s}$ denotes the range sampling frequency and $N_{r}$ denotes the number of range samples. In practice, (27) is realized by 1D sinc interpolation along the range direction. In the implementation, a fixed 8-point sinc interpolation kernel is used to evaluate the signal at the fractional index $n_{y}$.

After Stolt interpolation, an inverse Fourier transform is performed along the new range wavenumber dimension:

(29) $$S_{C}\left(\tau_{1},K_{x}\right)=F_{K_{y}}^{-1}\left\{S_{C}\left(K_{y},K_{x}\right)\right\}$$

where $F_{K_{y}}^{-1}\left\{\cdot \right\}$ denotes the inverse Fourier transform with respect to $K_{y}$, and $\tau_{1}$ denotes the new range time variable after the wavenumber remapping.

### 2.4. Azimuth Domain Postprocessing

After 2-D frequency domain focusing, the signal has been compressed in range and the range migration has been corrected by bulk compression and Stolt interpolation. However, due to the azimuth spectral reshaping introduced in the preprocessing stage, the focused signal is still represented on a scaled azimuth support. Therefore, azimuth domain postprocessing is required to restore the azimuth support and complete the final azimuth compression.

Let $K_{\mathrm{azp}}$ denote the equivalent azimuth chirp rate used in the azimuth postprocessing stage. The first azimuth postprocessing phase function is defined in the azimuth frequency domain as

(30) $$\Phi_{5}\left(f_{\eta_{1}}\right)=\exp\;\left(-j\frac{\pi }{K_{\mathrm{azp}}}f_{\eta_{1}}^{2}\right)$$

where $f_{\eta_{1}}$ is the azimuth frequency variable after azimuth preprocessing. Multiplying (30) and then performing an inverse Fourier transform along the azimuth frequency dimension gives

(31) $$S_{D}\left(\tau_{1},\eta_{2}\right)=F_{f_{\eta_{1}}}^{-1}\left\{S_{C}\left(\tau_{1},f_{\eta_{1}}\right)\;\Phi_{5}\left(f_{\eta_{1}}\right)\right\},$$

where $\eta_{2}$ denotes the scaled azimuth coordinate after the first azimuth inverse Fourier transform, and $F_{f_{\eta_{1}}}^{-1}\left\{\cdot \right\}$ denotes the inverse Fourier transform with respect to $f_{\eta_{1}}$.

To restore the azimuth support and complete the final azimuth compression, a second quadratic phase function is applied on the scaled azimuth coordinate:

(32) $$\Phi_{6}\left(\eta_{2}\right)=\exp\;\left(-j\frac{\pi }{K_{\mathrm{azp}}}\eta_{2}^{2}\right)$$

The final focused signal is then obtained by another inverse Fourier transform along the scaled azimuth coordinate:

(33) $$S_{\mathrm{img}}\left(\tau_{1},\eta_{3}\right)=F_{\eta_{2}}^{-1}\left\{S_{D}\left(\tau_{1},\eta_{2}\right)\;\Phi_{6}\left(\eta_{2}\right)\right\}$$

where $\eta_{3}$ denotes the final azimuth time coordinate.

The focused image is finally expressed in magnitude form as

(34) $$I\left(\tau_{1},\eta_{3}\right)=\left|S_{\mathrm{img}}\left(\tau_{1},\eta_{3}\right)\right|$$

## 3. GPU-Based SAR Imaging: Design and Implementation

This section presents the GPU implementation of the sliding spotlight ω-k imaging algorithm on the Jetson AGX Orin embedded platform [10]. It first analyzes the computational characteristics of the ω-k imaging procedure and the architectural constraints of Jetson AGX Orin, then presents the overall implementation framework, and finally introduces the key optimization strategies, including hybrid data partitioning, asynchronous pipeline execution, customized Stolt interpolation, and FFT-related data reordering elimination.

As formulated in Section 2, the sliding spotlight ω-k imaging procedure consists of several sequential processing stages, including range frequency preprocessing, azimuth spectral reshaping, two-dimensional wavenumber domain focusing, Stolt interpolation, and azimuth postprocessing. Although these stages have different signal-processing purposes, their computational patterns can be summarized into three major categories. First, range and azimuth FFT and IFFT operations are repeatedly used to transform the signal among the range frequency, azimuth frequency, and wavenumber domains. Second, pointwise complex multiplications are applied for range frequency compensation, azimuth phase correction, bulk compression, and postprocessing phase compensation. Third, Stolt interpolation performs nonuniform resampling along the range wavenumber dimension to correct the residual range migration and complete the wavenumber domain focusing.

These operations are highly structured and exhibit strong data-level parallelism over large two-dimensional SAR data arrays. FFT operations can be accelerated using batched GPU FFT libraries [20], phase compensation can be implemented as element-wise parallel kernels, and Stolt interpolation can be mapped to fine-grained CUDA threads where each thread computes one output sample [21]. Therefore, the algorithmic structure derived in Section 2 provides a natural basis for GPU acceleration. However, when applied to high-resolution sliding spotlight SAR data, these operations also introduce substantial memory traffic and large intermediate arrays, making memory layout, data partitioning, and pipeline execution critical to the overall implementation efficiency.

**Figure 3:**
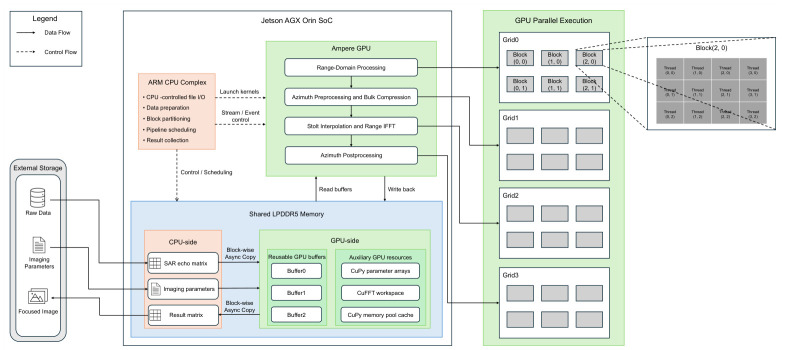
Technical architecture of the proposed GPU-oriented ω-k imaging framework on Jetson AGX Orin.

Figure 3 illustrates the integrated data flow and GPU implementation framework of the proposed method.

As shown in Figure 3, the proposed implementation maps the sliding spotlight ω-k imaging flow onto the heterogeneous architecture of Jetson AGX Orin. The ARM CPU complex performs CPU-controlled file I/O, data preparation, block partitioning, pipeline scheduling, and result collection. The raw echo data and imaging parameters are first loaded from external storage into CPU-side arrays in the shared LPDDR5 memory. During block-wise execution, the data are moved to GPU-side working buffers, where reusable buffers are cyclically used to support asynchronous pipeline execution.

The Ampere GPU reads data from the GPU-side buffers and executes the four main imaging stages, including range-domain processing, azimuth preprocessing and bulk compression, Stolt interpolation with range IFFT, and azimuth postprocessing. Auxiliary GPU resources, such as CuPy parameter arrays, cuFFT workspaces, and CuPy memory pool cache, are used to support FFT execution, phase compensation, and memory management [20,22]. The processed blocks are finally written back to the CPU-side result matrix and saved as the focused image. Solid arrows in the figure denote data movement, while dashed arrows denote control and scheduling flows, including kernel launches and stream/event control. The right side of the figure further illustrates that each imaging stage is mapped to GPU grids, blocks, and threads, thereby exposing the data-level parallelism required for efficient GPU acceleration.

### 3.1. Jetson AGX Orin Architecture and Performance Characteristics

Based on the data-parallel computational patterns discussed above, this work selects Jetson AGX Orin as the target embedded GPU platform. Jetson AGX Orin adopts a heterogeneous SoC architecture that integrates an ARM CPU subsystem, an Ampere GPU, and shared LPDDR5 memory [10]. This subsection analyzes the architectural characteristics that affect the proposed implementation.

**Figure 4:**
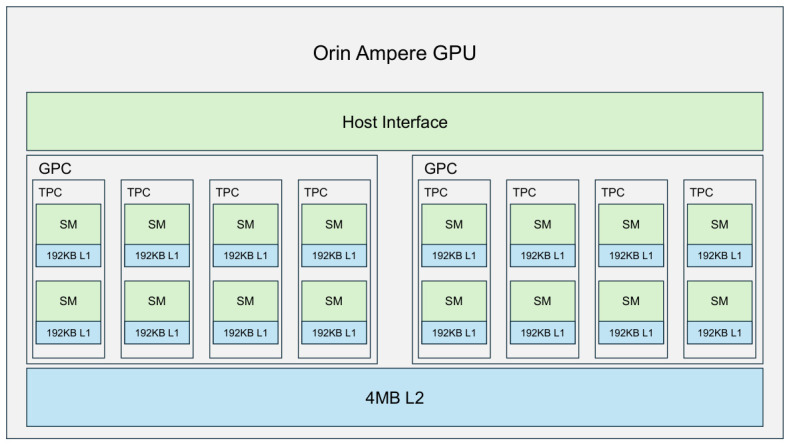
Orin Ampere GPU block diagram.

Jetson AGX Orin is a high-performance embedded computing platform developed by NVIDIA. It adopts a highly integrated System-on-Chip (SoC) architecture that combines a multi-core CPU, an Ampere-based GPU, and high-speed I/O interfaces on a single chip. The GPU architecture of Jetson AGX Orin is illustrated in Figure 4. The platform provides substantial parallel computing capability under a constrained power budget, making it suitable for embedded and onboard signal-processing applications [10,23]. In particular, its Ampere-based GPU contains multiple Streaming Multiprocessors (SMs) and a large number of CUDA cores, which provide the hardware basis for accelerating FFT operations, phase multiplications, and interpolation kernels in the proposed SAR imaging pipeline.

Although Jetson AGX Orin provides considerable parallel computing capability, its memory system is significantly different from that of data center GPUs. Data center GPUs usually rely on high-bandwidth memory, while Jetson AGX Orin adopts LPDDR5 memory and a unified memory architecture, where the CPU and GPU share the same physical memory space [10,23]. As a result, large-scale SAR imaging on this platform is strongly affected not only by arithmetic throughput, but also by memory bandwidth, data layout, and global memory traffic. For high-resolution sliding spotlight SAR imaging, the large matrix size and the alternating range–azimuth processing pattern can easily lead to non-coalesced memory accesses, full-matrix data movement, and repeated memory allocation overhead [24].

In terms of power consumption, Jetson AGX Orin is designed for embedded and edge computing scenarios. Its configurable power modes allow the system to operate under constrained power budgets, which is important for onboard processing applications where power supply, thermal dissipation, and physical size are limited [7,8,10]. Therefore, achieving efficient SAR imaging on this platform requires not only exploiting GPU parallelism, but also carefully reducing memory movement and improving sustained hardware utilization.

These architectural characteristics directly motivate the implementation strategies adopted in this work. The limited memory bandwidth motivates hybrid row-wise and column-wise data partitioning and memory access optimization. The throughput-oriented GPU architecture motivates fine-grained parallel execution for phase compensation and Stolt interpolation. In addition, the constrained memory capacity and power budget motivate reusable GPU buffers and asynchronous multi-stream pipeline execution to improve sustained GPU utilization while reducing memory management overhead. Based on these considerations, the following subsections introduce the proposed data partitioning strategy, asynchronous pipeline execution, customized Stolt interpolation kernel, and FFT optimization method.

### 3.2. Data Partitioning and Memory Access Optimization

As discussed in the previous subsection, the LPDDR5-based unified memory architecture of Jetson AGX Orin makes memory traffic a critical factor for large-scale SAR imaging. Therefore, the data partitioning strategy is designed to reduce unnecessary global data movement and improve memory locality during range- and azimuth-dominant processing stages.

In the considered ω-k imaging procedure, the computational workload alternates between the range and azimuth dimensions. Given a row-major data layout, only one dimension can be accessed in a fully coalesced manner, while accesses along the orthogonal dimension involve strided memory patterns. Consequently, when the dominant computation is performed along the non-contiguous dimension, the resulting non-coalesced memory accesses lead to underutilization of the available bandwidth. The alignment between computation direction and memory layout thus becomes a key factor in achieving high performance.

A straightforward approach to improving memory access locality is to perform an explicit global transposition of the data matrix so that the dominant computation direction aligns with the contiguous memory layout. However, for large-scale SAR data, such transposition requires a full read–write operation over the entire dataset, resulting in substantial memory traffic and additional storage overhead. More importantly, because the subsequent processing stages remain memory bandwidth-bound, the cost of this global data reorganization cannot be effectively amortized and may even degrade overall performance.

To address this issue, we propose a hybrid partitioning strategy to jointly optimize memory access locality and GPU memory utilization while maintaining computational efficiency. Instead of performing global data reorganization, the proposed method dynamically matches the partitioning direction to the dominant computation axis of each processing stage, thereby transforming a global, bandwidth-intensive reorganization problem into a series of localized block-level layout adjustments.

**Figure 5:**
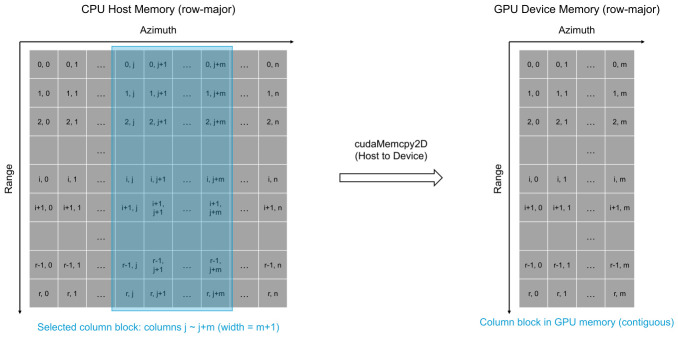
Column-wise blocking for range dominant processing. Each column block is reorganized into a contiguous GPU buffer to improve memory access efficiency for range direction operations.

Specifically, a combination of column-wise and row-wise blocking is employed. As illustrated in Figure 5, for operations dominated by range processing, column-wise partitioning is applied. The input matrix is divided into column blocks, and each block is moved into a contiguous GPU working buffer using a two-dimensional pitched memory copy, which efficiently supports non-contiguous column block access without inefficient column-by-column data movement. During this process, each block is reorganized directly into a contiguous device buffer without explicit intermediate transposition. Once transferred, all computations are performed on contiguous GPU buffers, improving memory access efficiency and overall computational performance. Within each block, rows represent range samples and columns represent azimuth samples in the corresponding sub-block, allowing range direction FFTs and element-wise operations to be executed efficiently without additional data rearrangement.

**Figure 6:**
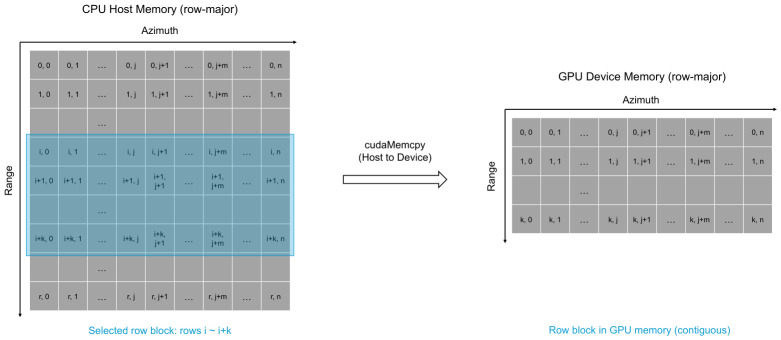
Row-wise blocking for azimuth dominant processing. Each row block is stored and processed as a contiguous GPU memory segment to improve memory access efficiency for azimuth direction operations.

Similarly, for operations dominated by azimuth processing, row-wise partitioning is adopted, as illustrated in Figure 6. In this case, the input matrix is divided into row blocks, each of which is transferred to the GPU as a contiguous memory segment. Since the row-wise layout is naturally aligned with the row-major storage format, the data within each block can be accessed in a fully coalesced manner without requiring additional data reorganization during transfer.

After being loaded into the GPU, computations are performed directly on contiguous row blocks, enabling efficient azimuth direction FFTs and element-wise operations. Within each block, rows correspond to range samples and columns correspond to azimuth samples in the local sub-block, preserving the original data organization while ensuring high memory access efficiency. This strategy effectively avoids the overhead associated with global data transposition and provides a computation-friendly data layout tailored to azimuth-dominant processing stages.

Overall, by dynamically switching between column-wise and row-wise partitioning strategies, the proposed method ensures that memory accesses remain largely contiguous for both range and azimuth dominant operations. This effectively improves global memory bandwidth utilization while avoiding the high overhead associated with global data transposition, making the approach particularly suitable for resource-constrained embedded GPU platforms such as the Jetson AGX Orin.

### 3.3. Asynchronous Multi-Stream Pipeline for Throughput Optimization

**Figure 7:**
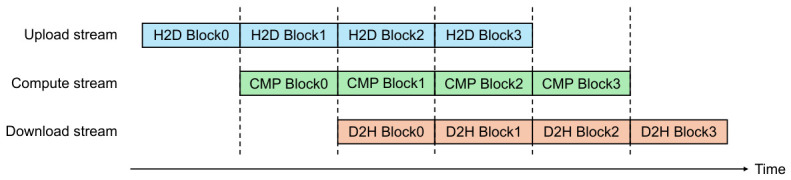
Three-stream pipelined execution strategy. After pipeline warm-up, data upload of the next block, GPU computation of the current block, and result download of the previous block are overlapped in steady state.

To further improve the end-to-end throughput of the proposed ω-k imaging pipeline on embedded GPUs, block-wise processing is combined with stream-level pipeline parallelism. As illustrated in Figure 7, the input data are divided into multiple blocks, and three non-blocking CUDA streams are used to organize data upload, GPU computation, and result download.

Instead of processing each block sequentially, the proposed pipeline overlaps the upload of the next block, the computation of the current block, and the download of the previous block. This design reduces idle intervals between consecutive blocks and improves sustained GPU utilization.

The detailed execution procedure of the proposed asynchronous pipeline is summarized in Algorithm 1.
**Algorithm 1** Asynchronous multi-stream pipeline pseudo-code  1:**Input:** input matrix *S*, processing stage *s*, block size *B*  2:**Output:** processed output matrix $S_{\mathrm{out}}$  3:// Determine block partitioning strategy.  4:Determine the dominant computation direction of stage *s*  5:**if** *s* is range-dominant **then**  6:    Partition *S* into column-wise blocks  7:    transfer_type = two-dimensional asynchronous memory copy  8:**else**  9:    Partition *S* into row-wise blocks10:    transfer_type = linear asynchronous memory copy11:**end if**12:// Initialize CUDA streams and reusable buffers.13:Create upload stream, compute stream, and download stream14:Preallocate $N_{\mathrm{buf}}$ reusable GPU buffers15:Create upload events $E^{\mathrm{up}}$ and compute events $E^{\mathrm{cmp}}$16:// Launch block-wise pipelined execution.17:**for** i=0 to $N_{b}-1$ **do**18:   // Select reusable buffer in a cyclic manner.19:   $b=i\;\mathrm{mod}\;N_{\mathrm{buf}}$20:   $G_{b}=$ reusable GPU buffer indexed by *b*21:   $B_{i}=$ the *i*-th input data block22:   // Upload current block.23:   Asynchronously upload $B_{i}$ to $G_{b}$ in the upload stream24:   Record upload event $E_{b}^{\mathrm{up}}$25:   // Compute current block after upload is complete.26:   Make the compute stream wait for $E_{b}^{\mathrm{up}}$27:   Execute GPU computation of stage *s* on $G_{b}$28:   Record compute event $E_{b}^{\mathrm{cmp}}$29:   // Download previous block while the next block is being uploaded and computed.30:   **if** i≥1 **then**31:       $p=\left(i-1\right)\;\mathrm{mod}\;N_{\mathrm{buf}}$32:       Make the download stream wait for $E_{p}^{\mathrm{cmp}}$33:       Asynchronously download the processed previous block from $G_{p}$ to $S_{\mathrm{out}}$34:   **end if**35:**end for**36:// Flush the pipeline by downloading the last processed block.37:**if** $N_{b}>0$ **then**38:     $l=\left(N_{b}-1\right)\;\mathrm{mod}\;N_{\mathrm{buf}}$39:     Make the download stream wait for $E_{l}^{\mathrm{cmp}}$40:     Asynchronously download the last processed block from $G_{l}$ to $S_{\mathrm{out}}$41:**end if**42:// Ensure all scheduled operations are completed.43:Synchronize upload stream, compute stream, and download stream44:**return** $S_{\mathrm{out}}$

For each processing stage, the partitioning direction is first determined according to the dominant computation axis. Range-dominant stages are processed using column-wise blocks and two-dimensional asynchronous memory copy, while azimuth-dominant stages use row-wise blocks and linear asynchronous memory copy. This allows the data transfer mode to match the storage pattern of each block.

As shown in Algorithm 1, the pipeline follows a producer–compute–consumer execution model coordinated by CUDA events. After a block is uploaded to a reusable GPU buffer, an upload event is recorded. The compute stream waits for this event before launching the corresponding GPU computation. After the computation finishes, a compute event is recorded, and the download stream waits for this event before transferring the processed block back to the host-side output buffer. In this way, data dependency is explicitly guaranteed while allowing different blocks to occupy different pipeline stages simultaneously.

A cyclic multi-buffer reuse mechanism is adopted to support the overlapped execution. For the *i*-th block, the buffer index is selected as $i\;\mathrm{mod}\;N_{\mathrm{buf}}$, so that a fixed number of GPU buffers can be reused across all blocks. This avoids frequent device memory allocation and deallocation during large-scale processing. In the implementation, three reusable buffers are used to match the three-stream pipeline, allowing the upload, computation, and download stages to operate concurrently after pipeline warm-up.

The asynchronous memory copy mode is selected according to the block layout. Column-wise blocks are transferred using two-dimensional asynchronous memory copy, which is suitable for non-contiguous host-side column blocks. Row-wise blocks are transferred using linear asynchronous memory copy because they are contiguous in row-major storage. After transfer, each block is stored in a contiguous GPU working buffer, which improves memory locality during FFTs, phase multiplications, and interpolation operations.

The peak GPU memory usage is controlled by preallocating a fixed number of reusable buffers instead of allocating memory for all blocks simultaneously. In the three-stream pipeline, only the blocks associated with upload, computation, and download are resident in GPU memory at the same time. The buffer index is selected cyclically as $i\;\mathrm{mod}\;N_{\mathrm{buf}}$, so the memory footprint of the working buffers remains constant regardless of the total number of blocks. This design prevents the multi-stream pipeline from increasing memory usage linearly with the number of blocks.

The block size is selected according to the available device memory and the temporary workspace required by each processing stage. A conservative safety factor is used so that the reusable working buffers, FFT workspaces, phase arrays, interpolation buffers, and input/output staging memory remain below the available GPU memory. In practice, the block size is bounded between predefined lower and upper limits to balance memory safety and kernel efficiency.

Overall, the proposed multi-stream pipeline transforms the conventional sequential block processing scheme into an overlapped data movement and computation workflow. By combining stage-aware block partitioning, event-based stream synchronization, and reusable GPU buffers, the implementation reduces data transfer overhead, improves sustained GPU utilization, and enhances the throughput of the ω-k imaging pipeline on the Jetson AGX Orin embedded platform.

### 3.4. GPU Implementation of Stolt Interpolation

In the ω-k imaging pipeline, Stolt interpolation constitutes one of the most computationally demanding stages due to its nonuniform resampling nature and intensive memory access. In this work, the interpolation is implemented on the GPU using a customized CUDA kernel, enabling efficient parallel execution over the range–azimuth grid.

The kernel adopts a two-dimensional thread mapping strategy, where each thread is responsible for computing a single output sample corresponding to a specific range–azimuth index. Specifically, the thread indices are directly mapped to the range and azimuth coordinates. This mapping avoids additional index decomposition, reduces integer arithmetic overhead, and naturally exposes fine-grained data-level parallelism.

To reduce computational overhead, the mapping from the wavenumber domain to the interpolation index is partially precomputed on the host side. The constant terms involved in the coordinate transformation are combined into scalar parameters and passed to the kernel. As a result, each thread only needs to evaluate the target interpolation position using a small number of floating-point operations, which reduces redundant computation and improves arithmetic efficiency.

The interpolation kernel is specialized for fixed 8-point sinc interpolation. By removing support for variable tap sizes, the implementation avoids unnecessary control logic and enables further compiler optimization. In addition, the fixed interpolation loop is explicitly unrolled using compiler directives, reducing loop overhead and improving instruction-level parallelism. The main procedure of the customized Stolt interpolation kernel is summarized in Algorithm 2.
**Algorithm 2** CUDA kernel pseudo code for fixed 8-point sinc Stolt interpolation  1:**Input:** input spectrum $S_{\mathrm{in}}$, range wavenumber vector $K_{r}$, azimuth wavenumber vector $K_{x}$, reference wavenumber $K_{rc}$, scaling factor α, half range size *h*, range size $N_{r}$, azimuth block width $N_{c}$, small threshold $\epsilon =10^{-6}$  2:**Output:** interpolated spectrum $S_{\mathrm{out}}$  3:// Calculate two-dimensional thread indices.  4:c=blockIdx.x×blockDim.x+threadIdx.x  5:r=blockIdx.y×blockDim.y+threadIdx.y  6:// Boundary check.  7:**if** $r\geq N_{r}$ or $c\geq N_{c}$ **then**  8:   **return**  9:**end if**10:// Compute Stolt interpolation position.11:$k_{r}=K_{r}\left[r\right]$12:$k_{x}=K_{x}\left[c\right]$13:$n_{y}=\left(\sqrt{k_{r}^{2}+k_{x}^{2}}-K_{rc}\right)\alpha +h$14:$n_{0}=n_{y}-1$15:// Initialize register accumulators.16:$a_{r}=0$17:$a_{i}=0$18:// Fixed 8-point sinc interpolation.19:**for** k=−3 to 4 **do**20:   $x=n_{0}+k$21:   $n_{k}=\mathrm{round}\left(x\right)$22:   $n_{k}=\mathrm{clamp}\left(n_{k},1,N_{r}\right)$23:   $\Delta =n_{0}-n_{k}$24:   // Compute sinc interpolation weight.25:   **if** |πΔ|<ϵ **then**26:       w=127:   **else**28:       w=sin(πΔ)/(πΔ)29:   **end if**30:   // ℜ{v} and ℑ{v} denote the real and imaginary parts of *v*.31:   // Load complex input sample and accumulate.32:   $v=S_{\mathrm{in}}\left(n_{k},c\right)$33:   $a_{r}=a_{r}+w\cdot ℜ\left\{v\right\}$34:   $a_{i}=a_{i}+w\cdot ℑ\left\{v\right\}$35:**end for**36:// Write back interpolated complex output sample.37:$S_{\mathrm{out}}\left(r,c\right)=a_{r}+ja_{i}$

Within each thread, the interpolation weights are computed on the fly. A small threshold condition is applied when evaluating the sinc function to avoid numerical instability near singular points. In the implementation, the threshold is set to $\epsilon =10^{-6}$. This threshold is used only to handle the removable singularity of the sinc function near zero. When |πΔ|<ϵ, the interpolation weight is assigned the limiting value w=1, which avoids division by a very small number without affecting the interpolation accuracy. The real and imaginary components of the interpolated result are accumulated in registers and written back to global memory only once, thereby minimizing global memory write operations.

It is worth noting that the input data are stored in row-major format, while the interpolation is performed along the range dimension. Therefore, each thread accesses neighboring samples along the range direction at a fixed azimuth index, resulting in a strided global memory access pattern with respect to the underlying storage layout. Although this limits memory coalescing efficiency, the impact is partially mitigated by the high degree of parallelism, the fixed lightweight computation per thread, and the reduced control overhead of the customized kernel.

The proposed CUDA implementation transforms Stolt interpolation into a fine-grained parallel operation. By combining two-dimensional thread mapping, host-side constant precomputation, fixed 8-point sinc interpolation, loop unrolling, register-level accumulation, and careful memory access design, the kernel achieves efficient execution and integrates seamlessly into the GPU-accelerated ω-k imaging pipeline.

### 3.5. FFT Optimization and Elimination of Data Reordering

Among the three computational patterns discussed at the beginning of this section, FFT and IFFT operations appear repeatedly across the range and azimuth processing stages. Therefore, reducing the auxiliary data movement associated with FFT execution is critical for overall performance.

FFT operations constitute a major computational and memory-intensive component in SAR imaging algorithms. In this implementation, both range and azimuth domain FFT/IFFT operations are executed on the GPU by invoking the cuFFT library through CuPy, enabling efficient large-scale parallel computation.

In conventional implementations, FFT operations are typically accompanied by the fftshift operation, whose purpose is to rearrange the spectrum by moving the zero-frequency component from the beginning of the array to its center. However, fftshift is essentially a global data reordering operation, which introduces several inefficiencies in high-performance computing environments, especially on GPUs.

First, fftshift requires explicit data movement across the entire array. This operation involves a full-array read and write, making it predominantly memory bandwidth-bound without contributing to computational acceleration. Second, the data reordering process leads to non-contiguous memory access patterns, which reduce memory coalescing efficiency and increase access latency. Third, fftshift is typically implemented as an independent kernel following the FFT, introducing additional kernel launch overhead and interrupting the computational pipeline.

To avoid these drawbacks, the proposed implementation replaces the explicit fftshift operation with an equivalent phase modulation strategy. Instead of physically rearranging the data, the same spectral centering effect can be achieved by multiplying the signal with a complex exponential phase term. For a discrete signal of length *N*, multiplying x[n] by ${\left(-1\right)}^{n}=\exp\left(j\pi n\right)$ in the time domain corresponds to a frequency domain shift of N/2 bins, which effectively centers the spectrum [25]. This effect is equivalent to that of fftshift under the periodicity of the discrete Fourier transform.

Based on this principle, the implementation applies the phase factor before and after the FFT operation to emulate the behavior of

fftshift(fft(fftshift(·)))

The same principle is also applied to centered IFFT operations by placing the corresponding phase modulation around the IFFT call. The entire process consists only of element-wise multiplications and FFT operations, without any explicit data movement or cross-thread communication. These element-wise operations are fully parallel and exhibit coalesced memory access on GPUs. As a result, the original memory-bound data reordering is transformed into a computationally efficient operation, which is significantly more suitable for GPU execution and leads to improved overall performance.

## 4. Results

To evaluate the effectiveness of the proposed ω-k imaging framework and its GPU-oriented implementation, a series of experiments were conducted on a Jetson AGX Orin embedded platform. The experiments aimed to assess both imaging quality and computational performance.

### 4.1. Simulation Results

To validate the imaging performance of the proposed method, a simulated sliding spotlight SAR dataset with point targets was used in the experiment. The main simulation parameters are listed in Table 1. The simulation scene was configured with high-resolution requirements in both range and azimuth directions, and the azimuth beam was steered during data acquisition to support sliding spotlight imaging.

**Figure 8:**
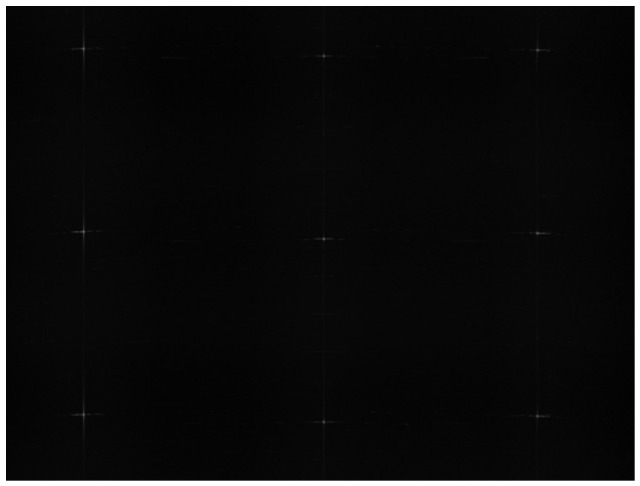
Imaging result of simulated point targets.

The imaging result of the simulated point target scene is shown in Figure 8.

The nine point targets are clearly focused and arranged in a regular 3 × 3 distribution. The global imaging result shows no obvious geometric distortion or defocusing, indicating that the proposed processing flow can effectively handle the range–azimuth coupling and spatially varying characteristics in the simulated sliding spotlight SAR data.

To further evaluate the focusing quality, three representative point targets located along the diagonal of the scene were selected and denoted as Target A, Target B, and Target C. The corresponding impulse response evaluation results are summarized in Table 2. The results show that the selected targets achieve consistent focusing performance in both range and azimuth directions. The resolution, PSLR, and ISLR values remain stable across different target positions, demonstrating that the proposed method provides good focusing uniformity over the simulated scene.

**Figure 9:**
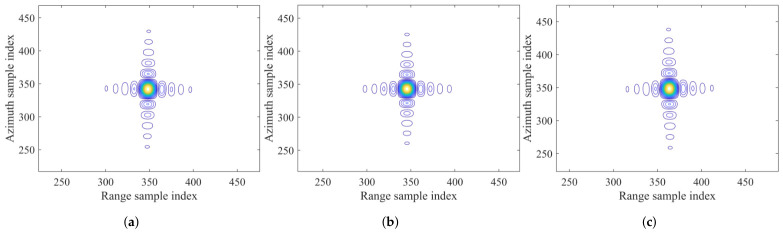
Impulse response results of the simulated point targets: (**a**) Target A; (**b**) Target B; (**c**) Target C.

Figure 9 shows the local impulse response results of the three selected point targets. The responses exhibit concentrated mainlobes and regular sidelobe patterns in both range and azimuth directions. Combined with the quantitative results in Table 2, the similar impulse response characteristics among the three targets indicate consistent focusing performance over different positions of the simulated scene. Overall, the simulated data experiment demonstrates that the proposed method can obtain well-focused SAR images under the given simulation configuration.

### 4.2. Implementation Performance on Jetson AGX Orin

The implementation performance was evaluated on the Jetson AGX Orin embedded platform. The input simulated SAR dataset had a size of 32,768 × 32,768, representing a large-scale imaging workload that imposes significant pressure on both memory capacity and memory bandwidth. For each configuration, the imaging procedure was executed multiple times, and the average execution time was used for performance comparison.

The end-to-end execution time was measured to evaluate the overall efficiency of the proposed implementation. The reported time includes the main processing stages of the ω-k imaging pipeline, including range- and azimuth-domain FFT/IFFT operations, phase compensation, Stolt interpolation, and data movement between host-side and GPU-side buffers involved in block-wise execution. For the 32,768 × 32,768 simulated SAR dataset, the proposed implementation achieved an end-to-end imaging time of 32.17 s on Jetson AGX Orin. This result shows the feasibility of executing the proposed large-scale ω-k imaging pipeline on the embedded Jetson AGX Orin platform.

To further analyze the contribution of each optimization strategy, an ablation study was performed on the 32,768 × 32,768 dataset. The standard implementation with global matrix transposition was used as the baseline to quantify the overhead of global data reorganization. The proposed hybrid data partitioning strategy was then enabled, replacing global transposition with data partitioning and memory access optimization. Based on this configuration, the asynchronous multi-stream pipeline, optimized GPU Stolt interpolation, and FFT optimization through the elimination of explicit data reordering were progressively enabled. The ablation results are summarized in Table 3. The speedup is calculated with respect to the baseline as

(35) $$\mathrm{Speedup}=\frac{T_{\mathrm{baseline}}}{T_{\mathrm{optimized}}},$$

where $T_{\mathrm{baseline}}$ denotes the execution time of the baseline and $T_{\mathrm{optimized}}$ denotes the execution time of each optimized configuration.

As shown in Table 3, replacing the standard global matrix transposition with the proposed hybrid data partitioning reduces the execution time from 80.28 s to 65.32 s. This result indicates that the proposed data partitioning strategy reduces global data movement and alleviates memory bandwidth pressure on Jetson AGX Orin. Based on this configuration, the asynchronous multi-stream pipeline further reduces the execution time to 49.71 s by overlapping data movement and computation. The optimized Stolt interpolation kernel improves performance by accelerating the nonuniform resampling stage, while the FFT optimization reduces overhead by eliminating explicit data reordering. Compared with the global transposition baseline, the full proposed implementation reduces the execution time from 80.28 s to 32.17 s, achieving a speedup of 2.50×. These results demonstrate that the proposed data partitioning, memory access, and pipeline optimizations are effective for large-scale SAR imaging on embedded GPU platforms such as Jetson AGX Orin.

## 5. Discussion

The experimental results demonstrate the effectiveness of the proposed GPU-oriented ω-k imaging framework for sliding spotlight SAR processing on an embedded platform. The simulated point target results show that the proposed method can obtain well-focused images, and the selected targets exhibit consistent impulse response characteristics in both range and azimuth directions. This indicates that the proposed processing flow can effectively handle the range–azimuth coupling and spatially varying phase characteristics of sliding spotlight SAR data.

From the implementation perspective, the results on Jetson AGX Orin verify the feasibility of executing large-scale ω-k imaging on an embedded GPU. For the 32,768 × 32,768 simulated SAR dataset, the proposed implementation achieves an end-to-end imaging time of 32.17 s. The ablation study further shows that the combination of asynchronous pipeline execution, optimized Stolt interpolation, and FFT optimization based on the elimination of explicit data reordering reduces the execution time from 80.28 s to 32.17 s, achieving a speedup of 2.50× over the baseline.

These results suggest that memory access efficiency is a key factor for high-resolution SAR imaging on embedded GPUs. Although the ω-k algorithm is highly parallelizable, its performance is strongly affected by memory bandwidth, data layout, and the alternating range–azimuth processing pattern. The proposed hybrid partitioning and pipeline execution strategies effectively improve memory locality and overlap data movement with computation, making the implementation more suitable for onboard platforms with limited resources.

The trade-off between focusing accuracy and execution time is mainly related to the Stolt interpolation stage. The proposed data partitioning, reusable buffer mechanism, asynchronous multi-stream pipeline, and FFT-related data reordering elimination only change the execution schedule and memory access pattern, without modifying the mathematical formulation of the ω-k imaging algorithm. Therefore, these optimizations reduce execution time without introducing additional focusing error. In contrast, the interpolation kernel length in the Stolt interpolation stage directly affects both resampling accuracy and computational cost. A longer sinc interpolation kernel can improve the approximation accuracy of nonuniform resampling, but it also increases memory accesses and arithmetic operations. A shorter kernel can reduce execution time, but may introduce interpolation error and slightly degrade the impulse response. In this work, an eight-point sinc interpolation kernel is adopted as a practical compromise between focusing accuracy and execution time on Jetson AGX Orin.

The present validation is based on simulated sliding spotlight SAR data generated under an ideal trajectory assumption. This setting is consistent with the main objective of this work, which is to develop and evaluate an embedded GPU implementation framework for large-scale sliding spotlight ω-k imaging, rather than to introduce a new motion compensation algorithm. Under the ideal trajectory model, the simulated point target results verify that the proposed processing chain can achieve accurate full-scene focusing for the designed 1 m resolution and 8 km scene extent. For practical airborne or spaceborne SAR data, non-ideal platform trajectories may introduce additional range errors and phase errors. The proposed framework can be extended by incorporating a motion compensation module before the ω-k focusing stage. Specifically, trajectory deviations can be estimated from navigation data or autofocus processing, and the corresponding motion-induced phase errors can be compensated during preprocessing. After motion compensation, the corrected echo data can be processed by the proposed ideal-trajectory ω-k imaging chain. Therefore, the current framework is compatible with non-ideal trajectory processing when an appropriate motion compensation front-end is included.

Nevertheless, the current experimental validation still has several limitations. First, the imaging experiments are mainly based on synthetic point target data. Although point target simulation is effective for evaluating impulse response, resolution, PSLR, ISLR, and spatial consistency, it cannot fully characterize possible artifacts in distributed scenes with textured scattering characteristics. Therefore, the current results should be interpreted as a verification of the correctness and efficiency of the proposed embedded GPU implementation under the simulated evaluation setting, rather than as a complete demonstration of practical achievability for all real SAR scenarios. Further validation using synthetic distributed scenes and measured sliding spotlight SAR data is needed to evaluate imaging artifacts, robustness, and practical applicability. Second, although embedded onboard processors are constrained by power consumption and thermal dissipation, the present work does not include a dedicated quantitative power or thermal profiling experiment. The experimental evaluation mainly focuses on imaging correctness, execution time, memory-aware implementation, and GPU acceleration on Jetson AGX Orin. The proposed strategies, including block-wise processing, reusable GPU buffers, reduced global data movement, and asynchronous pipeline execution, are designed to reduce unnecessary memory traffic and improve sustained hardware utilization, which is beneficial for power- and thermal-constrained execution. However, a complete evaluation of power consumption, temperature variation, and possible thermal throttling requires dedicated monitoring under controlled power modes and cooling conditions. Future work will investigate measured-data validation, distributed-scene simulation, adaptive block size selection, power and thermal profiling, mixed-precision computation, motion compensation, autofocus, and integration with onboard tasks such as target detection.

Overall, the proposed method provides a feasible solution for high-resolution sliding spotlight SAR imaging on embedded GPU platforms and offers a promising direction for onboard real-time SAR processing.

## Figures and Tables

**Table 1:** Simulation parameters of the SAR imaging experiment.

Parameters	Value
Center frequency	9.6 GHz
Pulse bandwidth	300 MHz
Center slant range	700 km
Range resolution	1 m
Range scene extent	8 km
PRF	5000 Hz
Initial azimuth scan angle	1.2°
Final azimuth scan angle	−1.2°
Azimuth resolution	1 m
Azimuth scene extent	8 km

**Table 2:** Impulse response evaluation results of simulated point targets.

Target	Range Direction			Azimuth Direction		
	Resolution (m)	PSLR (dB)	ISLR (dB)	Resolution (m)	PSLR (dB)	ISLR (dB)
Target A	0.72	−13.25	−10.25	0.66	−13.80	−11.64
Target B	0.72	−13.24	−10.15	0.65	−13.68	−11.11
Target C	0.72	−13.27	−10.26	0.66	−13.87	−11.61

**Table 3:** Ablation study of the proposed GPU optimizations on Jetson AGX Orin.

Configuration	Enabled Optimization	Execution Time (s)	Speedup
Baseline	Standard implementation with global matrix transposition	80.28	1.00×
+ Hybrid data partitioning	Replace global transposition with data partitioning and memory access optimization	65.32	1.23×
+ Multi-stream pipeline	Previous configuration + asynchronous multi-stream pipeline	49.71	1.61×
+ Stolt interpolation kernel	Previous configuration + optimized GPU Stolt interpolation	37.87	2.12×
+ FFT optimization	Full proposed implementation	32.17	2.50×

## Data Availability

The data used in this study were generated through numerical simulation. The simulation data and implementation code are available from the corresponding author upon reasonable request.
